# Prediction performance of serum placental growth factor (PLGF) human chorionic gonadotropin β (β-hCG) and PAPP-A levels in early pregnancy for pregnancy outcomes

**DOI:** 10.12669/pjms.38.7.5248

**Published:** 2022

**Authors:** Xiao Guo, Feng Wang, Jing Li, Zhankun Guo, Jing Wang

**Affiliations:** 1 Xiao Guo, Department of Obstetrics, Baoding Maternal and Child Health Hospital, Baoding 071000, Hebei, China; 2 Feng Wang, Dept. of Clinical Lab, Baoding Maternal & Child Health Hospital, Baoding 071000, Hebei, China; 3 Jing Li, Department of Obstetrics, Baoding Maternal and Child Health Hospital, Baoding 071000, Hebei, China; 4 Zhankun Guo, Department of Obstetrics, Baoding Maternal and Child Health Hospital, Baoding 071000, Hebei, China; 5 Jing Wang, Department of Obstetrics, Baoding Maternal and Child Health Hospital, Baoding 071000, Hebei, China

**Keywords:** Early pregnancy, PLGF, β-hCG, PAPP-A, Pregnancy outcome, Prediction performance

## Abstract

**Objectives:**

To investigate the prediction performance of serum placental growth factor (PLGF), free human chorionic gonadotropin β (β-hCG) and pregnancy-associated plasma protein A (PAPP-A) levels in early pregnancy for pregnancy outcomes.

**Methods:**

A total of 4256 pregnant women who underwent obstetric examinations in our hospital from June 2018 to June 2020 and completed their full pregnancy were included in the study. The clinical pregnancy outcomes of pregnant women with different PLGF, PAPP-A and β-hCG levels in early pregnancy were recorded, and the prediction performance of the above indicators for adverse pregnancy outcomes was discussed.

**Results:**

Pregnant women with increased or decreased PLGF or increased PAPP-A or β-hCG had significantly higher incidence of adverse pregnancy outcomes than normal pregnant women. Pregnant women with abnormal pregnancy had significantly higher β-hCG and PLGF, and lower PAPP-A than those with normal pregnancy. The sensitivity of serum PLGF, β-hCG and PAPP-A in early pregnancy for predicting adverse pregnancy outcomes was 95.13%, 94.19% and 97.75%, and the specificity was 84.31%, 85.80% and 83.22%, respectively.

**Conclusions:**

Serum PLGF, PAPP-A and β-hCG in early pregnancy are more effective in predicting adverse pregnancy outcomes. Clinical monitoring of patients with increased PLGF, decreased PAPP-A, and increased β-hCG should be strengthened, especially the monitoring of antepartum examination and B-ultrasound detection of pregnant women with abnormal indicators in middle and late pregnancy, so as to identify adverse pregnancy outcomes as early as possible and give targeted intervention.

## INTRODUCTION

Pregnancy outcomes may be significantly affected by delayed clinical interventions for preeclampsia (PE), gestational diabetes mellitus (GDM), and intrauterine fetal growth retardation (FGR).^1^-^3^ Adverse pregnancy outcomes pose a serious threat to maternal and fetal health, but in the study of patients with adverse pregnancy outcomes, there is no precise clinical prediction index for disease monitoring and prevention.^4^ Therefore, priority should be given in clinical studies to select scientific and effective serological indicators for predicting pregnancy outcomes. Beta Human Chorionic Gonadotropin (β-hCG), secreted by embryonic trophoblast syncytial cells, is the first effective signal for the embryo or fetus to be released into the mother, and it runs through the entire pregnancy process.^5^

Pregnancy-associated plasma protein A (PAPP-A) and placental growth factor (PLGF) can predict PE. However, healthy pregnant women have a wide range of PLGF, and their individual differences affect the predictive value of PLGF and PAPP-A single indicators.^6^,^7^ In this study, a retrospective analysis was conducted on the general clinical data of ten thousand patients with early pregnancy examination, and their pregnancy outcomes were recorded during follow-up to analyze the prediction performance of the above indicators in adverse pregnancy outcomes.

## METHODS

### General Information

A retrospective cohort study was conducted to follow up the general clinical data of 4,256 pregnant women collected from our hospital’s electronic medical record system from June 2018 to June 2020 who had undergone an obstetric examination in our hospital and completed the complete pregnancy process. All the pregnant women were 23-38 years old, with an average age of (30.56±3.14) years, and a gestational age of 11-13+6 w, with an average gestational age of (12.05±0.54) week.

### Ethical Approval

The study was approved by the Institutional Ethics Committee of Baoding Maternal and Child Health Hospital on February 6, 2018 (No.2018[68]), and written informed consent was obtained from all participants

### Inclusion criteria:

- Pregnant women with singleton pregnancy;
- Pregnant women with complete clinical data
- Pregnant women with normal antepartum examination before enrollment and without threatened abortion.

### Exclusion criteria:

- Pregnant women with ectopic pregnancy or hydatidiform mole;
- Pregnant women with uterine fibroids, ovarian cysts, and uterine malformations;
- Pregnant women with abnormal blood coagulation function;
- Pregnant women with poor life history;
- Pregnant women with severe infectious diseases.

### Serological examination in early pregnancy

After the pregnant women were enrolled, their peripheral blood and fasting venous blood were collected and centrifuged at a rate of 3,000 r/min for 10 minutes, and the supernatant was taken for test. The serum β-hCG level of pregnant women was detected by ELISA, and all β-hCG human ELISA kits used were purchased from Kmaels (Shanghai) Biotechnology Co., LTD. PAPP-A and PLGF levels were detected by the Wallac AutoDELFIA 1235 automatic time-resolved fluorescence immunoassay, and all PAPP-A and PLGF kits used were purchased from Perkin Elmer. All procedures were performed in strict accordance with the kit instructions, and subjects were ordered to undergo regular antepartum examination. The gestational age was estimated based on the double parietal-diameter results of fetal B-ultrasound, and the concentration of serologic markers was transformed into multiple of the medion (MOM) according to the body weight information and the age of the pregnant woman. The normal range of each indicator was 0 < β-hCG < 3 IU/L, 0.42 < PAPP-A < 2.5 MOM, 0 < PLGF < 87 μg/L.

### Statistical Analysis

SPSS 20.0 software was utilized to process the data. The measurement data were represented by (*x̅*±*s*), and *t* test was performed. Moreover, the receiver operating characteristic curve (ROC) was drawn to evaluate the predictive effect of each index on adverse pregnancy outcomes. *P*<0.05 indicates a statistically significant difference.

## RESULTS

Among 4256 pregnant women, 3515 (82.59%) had normal outcomes, and 741 (17.41%) had adverse outcomes. Among them, the incidence rates of structural malformations, preterm delivery, hypertension, preeclampsia, hypothyroidism and placenta previa in early pregnancy were 1.06%, 3.31%, 2.33%, 2.30%, 1.90% and 2.33%, respectively, as shown in Table-I.

**Table I:** Comparison of pregnancy outcomes.

Pregnancy outcome	Single disease	Single disease	Single disease (%)
Normal	-	-	3515 (82.59)
Fetal abnormalities			
T21	3	0	3 (0.07)
T18	1	0	1 (0.02)
Other chromosomal abnormalities	3	0	3 (0.07)
Aploidy and open neural tube defects	1	0	1 (0.02)
Fetal demise	20	3	23 (0.54)
Structural malformations	39	6	45 (1.06)
Premature delivery	110	31	141 (3.31)
Small-for-gestational age infant	26	5	31 (0.73)
Maternal abnormality			
Hypertension	87	12	99 (2.33)
Preeclampsia	86	12	98 (2.30)
Hyperthyroidism	35	1	36 (0.85)
Hypothyroidism	68	13	81 (1.90)
Uterine malformation	18	4	22 (0.52)
Cholestasis during pregnancy	22	8	30 (0.70)
Placental abnormalities			
Accreta/adhesion	18	10	28 (0.66)
Placenta previa	82	17	99 (2.33)

The incidence of adverse pregnancy outcomes in PLGF or PPAP-A increased group or PPAP-A decreased group or β-hCG decreased group was significantly higher than that in the normal control group (P<0.05), as shown in Table-II.

**Table II:** Comparison of adverse maternal pregnancy outcomes with abnormal serum markers in early pregnancy [n (%)].

Pregnancy outcome	Number of cases	Serum index normal control group	Reduced PLGF	Increased PLGF	Reduced PAPP-A	Increased PAPP-A	Reduced β-hCG	Increased β-hCG
All outcomes	4256	3977 (93.44)	45 (1.06)	38 (0.89)	47 (1.11)	48 (1.13)	49 (1.15)	52 (1.22)
Normal outcomes	3515	3297 (82.90)	42 (93.33)	27 (71.05)	33 (70.21)	39 (81.25)	38 (77.55)	33 (63.46)
Mother								
Hypertension	99	89 (2.24)	1 (2.22)	2 (5.26)	2 (4.26)	2 (4.17)	2 (4.08)	1 (1.92)
Preeclampsia	98	90 (2.26)	0 (0)	1 (2.63)	2 (4.26)^*^	1 (2.08)	1 (2.04)	3 (5.77)^*^
Early-onset	10	4 (0.10)	0 (0)	0 (0)	2 (4.26)^*^	0 (0)	1 (2.04)	3 (5.77)^*^
Late-onset	88	78 (1.96)	1 (2.22)	1 (2.63)	1 (2.13)	2 (4.17)	2 (4.08)	3 (5.77)
Hyperthyroidism	36	32 (0.80)	0 (0)	2 (5.26)	1 (2.13)	1 (2.08)	0 (0)	1 (1.92)
Hypothyroidism	81	76 (1.91)	0 (0)	1 (2.63)	1 (2.13)	0 (0))	2 (4.08)	2 (3.84)
Cholestasis during pregnancy	30	25 (0.63)	0 (0)	1 (2.63)	1 (2.13)	1 (2.08)	1 (2.04)	1 (1.92)
Uterine malformation	22	18 (0.45)	0 (0)	2 (5.26)^*^	0 (0)	0 (0)	0 (0)	2 (3.84)^*^
Placenta accreta/adhesion	28	23 (0.58)	0 (0)	1 (2.63)	1 (2.13)	1 (2.08)	1 (2.04)	1 (1.92)
Placenta previa	99	87 (2.19)	1 (2.22)	1 (2.63)	3 (6.38)	1 (2.08)	1 (2.04)^*^	5 (9.62)^*^

***Note:***

* P<0.05 compared with the control group

**Fig.1:**
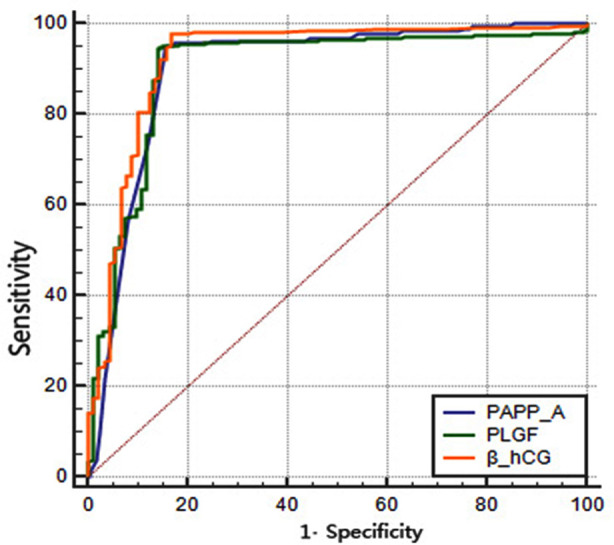
Prediction performance of serum PLGF, β-hCG and PAPP-A levels in early pregnancy for adverse pregnancy outcomes.

The incidence of fetal adverse outcomes in pregnant women with increased PLGF or increased PAPP-A or decreased PAPP-A or decreased β-hCG was significantly higher than that in the normal control group (P<0.05), as shown in Table-III. β-hCG and PLGF in pregnant women adverse pregnancies were significantly higher than those with normal pregnancies, while PAPP-A was lower than that with normal pregnancies, as shown in Table-IV. The sensitivity of early serum PLGF, β-hCG and PAPP-A in predicting adverse pregnancy outcomes was 95.13%, 94.19% and 97.75%, and the specificity was 84.31%, 85.80% and 83.22%, respectively, showing favorable performance, as shown in Table-V and Fig.1.

**Table III:** Comparison of adverse pregnancy outcomes of fetuses with abnormal serum markers in early pregnancy [n (%)].

	Number of cases	Normal control group	Reduced PLGF	Increased PLGF	Reduced PAPP-A	Increased PAPP-A	Reduced β-hCG	Increased β-hCG
All outcomes	4256	3977 (93.44)	45 (1.06)	38 (0.89)	47 (1.11)	48 (1.13)	49 (1.15)	52 (1.22)
Normal outcomes	3515	3297 (82.90)	42 (93.33)	27 (71.05)	33 (70.21)	39 (81.25)	38 (77.55)	33 (63.46)
Fetus								
Chromosome abnormality	7	3 (0.08)	0 (0)^*^	1 (2.63)^*^	1 (2.13)^*^	1 (2.08)^*^	0 (0)	1 (1.92)^*^
Structural malformations	45	41 (1.03)	1 (2.22)	0 (0)	1 (2.13)	0 (0)	2 (4.08)	0 (0)
Fetal demise	23	11 (0.28)	0 (0)^*^	2 (5.26)^*^	2 (4.26)^*^	2 (4.17)^*^	2 (4.08)^*^	4 (7.69)^*^
<28w	16	7 (0.18)	0 (0)^*^	1 (2.63)^*^	1 (2.13)^*^	2 (4.17)^*^	2 (4.08)^*^	3 (5.77)^*^
>28w	7	1 (0.03)	0 (0)	1 (2.63)^*^	1 (2.13)^*^	1 (2.08)^*^	0 (0)^*^	3 (5.77)^*^
Premature delivery	141	127 (3.19)	1 (2.22)	3 (7.89)	2 (4.26)	2 (4.17)	2 (4.08)	4 (7.69)^*^
>34w	125	116 (2.92)	1 (2.22)^*^	1 (2.63)	1 (2.13)	1 (2.08)	1 (2.04)	4 (7.69)^*^
≤34w	16	15 (0.38)	0 (0)	0 (0)	1 (2.13)	0 (0)	0 (0)	0 (0)
Small-for-gestational age infant	31	27 (0.68)	0 (0)	1 (2.63)	2 (4.26)^*^	0 (0)	1 (2.04)^*^	0 (0)
<P_3_	12	9 (0.23)	0 (0)	1 (2.63)	1 (2.13)	0 (0)	1 (2.04)	0 (0)
P_3_~P_10_	19	18 (0.45)	0 (0)	0 (0)	1 (2.13)	0 (0)	0 (0)	0 (0)

***Note:***

* P<0.05 compared with the control group.

**Table IV:** Comparison of serum PLGF, β-hCG and PAPP-A levels in pregnant women with different pregnancy outcomes in early pregnancy (*x̅*±*s*)

Group	Number of cases	β-hCG (IU/L)	PAPP-A (MoM)	PLGF (μg /L)
Normal pregnancy	3515	2.15±0.14	1.31±0.15	68.11±7.29
Adverse pregnancy	741	5.94±0.61	0.29±0.03	107.26±10.25
T		329.599	184.313	122.827
P		<0.001	<0.001	<0.001

**Table V:** Prediction performance of serum PLGF, β-hCG and PAPP-A levels in early pregnancy for adverse pregnancy outcomes.

Indicators	Sensitivity (%)	Specificity (%)	AUC	AUC cutoff value
PLGF	95.13	84.31	0.900	1.24
β-hCG	94.49	85.80	0.898	103.25
PAPP-A	97.75	83.22	0.922	5.23

## DISCUSSION

Blood tests in early pregnancy can screen whether pregnant women suffer from high-risk such as diabetes, hypothyroidism, so that early intervention and treatment measures can be taken immediately.^8^ In this study, the incidence of structural malformations, preterm delivery, hypertension, preeclampsia, hypothyroidism, and placenta previa were relatively high in early pregnancy. Without timely intervention, the incidence of adverse pregnancy outcomes may increase as the pregnancy progresses. Therefore, strengthening serological screening in early pregnancy is expected to be the key to improving pregnancy outcome.

PLGF is a vascular endothelial growth factor secreted by syncytiotrophoblast, which can promote trophoblast migration, proliferation and induce angiogenesis, and is closely related to vascular recasting.^9^-^11^ It was found in this study that the incidence of uterine malformations, chromosomal abnormalities, structural malformations and fetal demise in the increased PLGF group was significantly higher than that in the normal PLGF group, while the incidence of chromosomal abnormalities, fetal demise below 28 weeks and premature delivery after 34 weeks in the decreased PLGF group was significantly higher than that in the normal control group. According to analysis, PLGF can promote cell mitosis to a certain extent. The incidence of chromosomal abnormalities, structural malformation and fetal loss will be significantly increased after the occurrence of horizontal abnormalities.^12^-^13^

It was proposed in a study by Fruscalzo et al.^14^ that PAPP-A level changes may also occur in some pregnancy complications, such as placental abruption, spontaneous abortion, small-for-gestational age, premature rupture of membranes, stillbirth, preeclampsia, and fetal growth retardation suggesting that PAPP-A level changes have important scientific value in pregnation-related studies.^15^ This study showed that the total incidence of preeclampsia and the incidence of early-onset preeclampsia, chromosome abnormality, fetal demise and small-for-gestational age infants were significantly higher in the decreased PAPP-A group than in the normal PAPP-A group, and the incidence of chromosome abnormality and fetal demise was significantly higher in the increased PAPP-A group than in the normal control group, indicating that PAPP-A abnormality will increase the incidence of preeclampsia, chromosomal abnormalities, fetal demise, and small-for-gestational age infants.^16^

hCG is the earliest glycoprotein hormone found in the maternal placenta, in which β subunit has strong specificity and is mainly synthesized and secreted by placental syncytic trophoblast cells, and the maternal and placental trophoblasts are directly connected.^17^ Studies have proposed that β-hCG is a pregnancy-specific marker, which can effectively reflect the function of trophoblasts in the placenta of pregnant women.^18^ It was found in this study that the incidence of early-onset preeclampsia, uterine malformations, incidence, placental previa, chromosomal abnormalities, fetal demise, premature delivery, and premature delivery after 34w in the increased β-hCG group was significantly higher than that of normal β-hCG group. The incidence of placenta previa, fetal demise, and small-for-gestational age infants in the decreased β-hCG group was significantly higher than that of the normal β-hCG group, suggesting that abnormal β-hCG levels may increase preeclampsia, uterine malformations, incidence, uterine malformation, placenta previa, chromosomal abnormalities, fetal demise, premature delivery, and small-for-gestational age infants. After ischemia and hypoxia, local syncytial trophoblasts in the placenta tissue will become degenerate and necrotic, affecting β-hCG secretion and leading to spontaneous abortion after luteal hypoplasia.^19^ Moreover, the concentration of β-hCG significantly increased under physiological conditions, which is often used in the diagnosis of early pregnancy.^20^

In this study, β-hCG and PLGF of pregnant women with adverse pregnancy were significantly higher than those with normal pregnancy, and PAPP-A was lower than those with normal pregnancy. The ROC curve showed that the early examination of serum PLGF, β-hCG, and PAPP-A had better prediction performance in adverse pregnancy outcomes, suggesting that that pregnant women with high β-hCG, high PLGF and low PAPP-A in early pregnancy should be followed up and targeted intervention should be given to improve pregnancy outcomes.

### Limitations of this study

This was a retrospective study, with limited clinical data available and limited follow up. Further intervention trials are needed in the future to confirm these results.

## CONCLUSION

To put it in a nutshell, PLGF, β-hCG, and PAPP-A have certain prediction performance in adverse pregnancy outcomes. Clinical monitoring of patients with increased PLGF, decreased PAPP-A, and increased β-hCG should be strengthened to identify adverse pregnancy outcomes as early as possible and give targeted intervention.

### Authors’ Contributions:

**XG & FW:** Designed this study, prepared this manuscript, are responsible and accountable for the accuracy and integrity of the work. **JL & ZG:** Collected and analyzed clinical data. **JW:** Data analysis, significantly revised this manuscript.
